# Contributions to early HIV diagnosis among patients linked to care vary by testing venue

**DOI:** 10.1186/1471-2458-8-220

**Published:** 2008-06-25

**Authors:** Michael S Lyons, Christopher J Lindsell, DeAnna A Hawkins RN, Dana L Raab RN, Alexander T Trott, Carl J Fichtenbaum

**Affiliations:** 1 Department of Emergency Medicine, University of Cincinnati College of Medicine, Cincinnati, Ohio USA; 2 Infectious Disease Center, University of Cincinnati College of Medicine, Cincinnati, Ohio USA

## Abstract

**Objective:**

Early HIV diagnosis reduces transmission and improves health outcomes; screening in non-traditional settings is increasingly advocated. We compared test venues by the number of new diagnoses successfully linked to the regional HIV treatment center and disease stage at diagnosis.

**Methods:**

We conducted a retrospective cohort study using structured chart review of newly diagnosed HIV patients successfully referred to the region's only HIV treatment center from 1998 to 2003. Demographics, testing indication, risk profile, and initial CD4 count were recorded.

**Results:**

There were 277 newly diagnosed patients meeting study criteria. Mean age was 33 years, 77% were male, and 46% were African-American. Median CD4 at diagnosis was 324. Diagnoses were earlier via partner testing at the HIV treatment center (N = 8, median CD4 648, p = 0.008) and with universal screening by the blood bank, military, and insurance companies (N = 13, median CD4 483, p = 0.05) than at other venues. Targeted testing by health care and public health entities based on patient request, risk profile, or patient condition lead to later diagnosis.

**Conclusion:**

Test venues varied by the number of new diagnoses made and the stage of illness at diagnosis. To improve the rate of early diagnosis, scarce resources should be allocated to maximize the number of new diagnoses at screening venues where diagnoses are more likely to be early or alter testing strategies at test venues where diagnoses are traditionally made late. Efforts to improve early diagnosis should be coordinated longitudinally on a regional basis according to this conceptual paradigm.

## Background

The HIV epidemic continues despite decades of aggressive prevention efforts and advances in effective treatment. An estimated one-quarter of those infected remain unaware of their HIV status, and late diagnosis remains common. [1-3] Traditional HIV screening that relies on resource-intensive outreach, or individuals to seek testing, has failed to fully access at-risk populations. Early diagnosis is essential to enable opportunities to halt further transmission [4] and to obtain medical therapy that improves morbidity and mortality for infected patients. [5] The CD4+ T cell count is the accepted marker of disease progression, and is a surrogate measure of how early in the disease the diagnosis has been made. [2,3,6,7] CD4+ count declines variably in HIV patients but typically decreases by an average of 60–100/μL per year [8-10].

Strategies to improve early diagnosis necessarily depend on who is tested and under what circumstances. Generally, later diagnosis is associated with diagnostic testing as a result of illness, while earlier diagnosis is more typical of patient self-request for testing, self-perceived risk, or universal screening. [2,11] Modifications to expand universal screening within health care settings have been proposed. [12] The CDC initiative particularly encourages screening in non-traditional settings such as emergency departments, substance abuse treatment centers, and corrections facilities. [7,11-14] The resources required to implement these changes broadly have not yet been allocated.

Understanding the differences between test venues in terms of numbers of new diagnoses successfully linked to care and the extent to which those diagnoses are early or late would inform health care planners on current practice patterns and resource use. For example, patients who are seeking HIV testing may self-refer to different venues than patients who are seeking medical care because of symptoms. Prior reports have examined the contributions by different screening venues to HIV testing. [1,7,13] However, to our knowledge, variation in stage of diagnosis by screening venue has not been assessed. To improve rates of early HIV diagnosis on a regional basis, policy makers would benefit from an improved understanding of diagnosis patterns to inform future allocation of scarce prevention resources.

We hypothesized that diverse regional testing venues would vary significantly in the number of new diagnoses made with successful linkage to care and the initial CD4 count of those patients at the time of diagnosis. This study was designed with the following two objectives, 1) to determine how many newly diagnosed persons were successfully referred by each local test venue relative to the total number of newly diagnosed persons seen at the regional HIV treatment center, and 2) to assess the differences in initial stage of HIV by test venue. We were specifically interested in determining the relative contribution of a publicly funded emergency department testing program to regional patterns of diagnosis.

## Methods

### Study Design

This was a retrospective cohort study of newly diagnosed HIV infected persons successfully referred to a regional infectious disease treatment center between July 1, 1998 and June 31, 2003. The local Institutional Review Board approved the study.

### Setting

Subjects were identified from lists of new patients visiting the region's only dedicated provider of HIV/AIDS care, which is located on the campus of an academic hospital in an urban area. This setting is also uniquely characterized by a highly active emergency department HIV screening program within the university hospital proximate to the HIV treatment center. [15,16] The surrounding metropolitan statistical area of approximately 2 million has an AIDS case rate of 5.7 per 100,000 (2005, HIV Surveillance report). The surrounding county has a population of 806,652 that is 25% African-American and 1.5% Hispanic.(2005, US Census)

### Source Population

The HIV treatment center, called the Infectious Diseases Center (IDC), provides the vast majority of primary and consultative care to HIV and AIDS patients in the region. In 2005, there were 2184 patients known to be living with HIV/AIDS in the Cincinnati Metropolitan Area (Hamilton, Butler, Warren, Clermont Counties). [17] There were an estimated additional 245 living in Northern Kentucky and Western Indiana. Of these not all are in care continuously. In 2007, the IDC saw 1626, or about 67% of those known to be living with HIV/AIDS in the region. Of the remainder, the IDC estimates that 300 are seen by others and 500 are not in care. Overall, it is estimated that the IDC provides care to about 85% of all HIV and AIDS patients known to be in care in this region.

Publically available surveillance data on CD4 count at diagnosis testing venue and facility of diagnosis for the Cincinnati region are provided by the IDC. The IDC provides these data to the Ohio Department of Health and the local health department does not systematically collect any data not reported by the IDC. These data are not publicly reported by the Ohio Department of Health.

### Selection of participants

Records for all patients newly referred to the regional HIV treatment center during the study period were screened for inclusion. These logs were created prospectively as new patients were received by the clinic, but they did not differentiate newly referred patients from the subset that were newly diagnosed. Any person that moves to the region and seeks care at the IDC is given a new patient appointment and logged as a new patient. In addition, if a patient had been seen previously but had been lost to follow up for 2 years or more, they were similarly considered a new patient. Thus, the list of new patients maintained by the IDC contains many patients who have HIV but are not newly diagnosed.

Patients were included in the study if: 1) their complete medical record could be located, 2) there was a clinic note for the first visit, 3) the first visit was within the specified study period, 4) the patient was HIV positive, and 5) there was no evidence that the patient had been aware of their diagnosis more than two months prior to the first visit. Patient awareness of a prior HIV positive diagnosis was defined as any acknowledgment of previous treatment, treatment refusal, or a clear statement in the medical record suggesting that the patient was aware of their diagnosis. Patients were excluded if there was no CD4 evaluation within 60 days of their initial HIV treatment center appointment.

### Data collection

A standardized case report form was used for data abstraction. Data collected included: 1) patient demographics, 2) initial CD4 count and viral load within 60 days of initial visit, 3) venue in which HIV testing was initially done, 4) reason for HIV testing if noted, and 5) risk factors, symptoms, and signs of HIV recorded at the initial visit. Explicit *a priori *definitions for abstraction and coding of all data elements were developed and emphasized during training of chart abstractors. Variable definitions were not amended after study initiation. Missing data points were noted as such after confirmation by a dual abstraction process.

Chart abstraction used a two stage methodology including screening for study enrollment followed by formal chart abstraction. For screening, three clinical study assistants were trained to review intake visit documentation. The training period was concluded when study assistants could classify charts accurately according to inclusion and exclusion criteria. Each determination of eligibility was confirmed by the study coordinator until competence was achieved. At this stage, reviewers were liberal with inclusion; when any doubt existed, the patient was included. To verify exclusions based on medical records not being available, charts for each patient were sought on two separate occasions by two different investigators before being declared unavailable. Records for every patient included by the clinical study assistant were secondarily reviewed by trained research nurses to confirm inclusion criteria and complete primary data abstraction.

There were 4 research nurses, each with a minimum of 5 years of clinical experience, selected to conduct chart abstraction. To avoid bias, research nurses were not previously associated with the HIV treatment center or emergency department screening program, and were blinded to the study hypothesis. The abstractors were familiarized with the general format and terminology of the HIV treatment center records with the assistance of the center staff. Chart abstractors were trained in the study specific chart abstraction methods and data definitions prior to data collection by the study investigators. The abstractors began training by abstracting a series of 5 practice charts, which were then checked for accuracy both by duplicate abstraction and by investigator review. This process was repeated serially until approximately 90% accuracy was consistently achieved. Monitoring for data quality used independent dual abstraction of each record with adjudication of any discrepancies by one of the investigators. Concordance between abstractors was not statistically assessed. Ongoing education was conducted whenever discrepancies occurred to minimize error rate through a continuous feedback mechanism.

### Outcome measures

The main measures are the initial CD4 count and referral source. Testing venues were grouped according to the referral center's understanding of each facility's patient population, funding sources, and testing methods. Medical testing venues were classified to include inpatient (hospital), outpatient (primary provider or clinic), emergency departments without dedicated testing programs, institutional testing done in prisons, substance abuse, and mental health centers, and medical testing of any unknown source. Public health testing included the local health department, Planned Parenthood and a local HIV/AIDS advocacy, prevention and case-management organization, which were all funded by the local health department to conduct HIV testing. Much of the testing by the health department and publically funded testing agencies was either due to self-referral by patients or conducted as part of evaluation for sexually transmitted infections or pregnancy. Other venues not classified as health care or public health testing included a health department funded HIV counseling and testing program in an emergency department [15,16], a publicly funded partner testing program at the region's HIV treatment center, and universal screening by the blood bank, military, and insurance pre-screening. Patients identified outside of the region or with an unknown referral source were categorized separately. Secondary measures included risk behavior profile and the reason for the testing that lead to diagnosis, if noted.

### Primary Data Processing and Analysis

Completed and adjudicated screening forms and case report forms were entered in duplicate into electronic format for subsequent analysis. Prior to analysis, data underwent a comprehensive cleaning process; each case was tested for internal consistency and incompatible data elements were referred back to an independent chart abstractor for revision or confirmation. Examples of incompatible data include potentially inconsistent risk factor information such as a male patient self reporting sex with men as a risk factor but who also identified as a heterosexual male. Missing data that could not be located were declared missing for analysis and are reported as such.

Summary statistics are used to report the data, medians and ranges are used for continuous variables and frequencies and proportions are used for categorical variables. CD4 counts were compared between testing venues using the Mann-Whitney U test. Analyses were conducted using SPSS v15.0.1 (SPSS Inc., Chicago, IL) and Microsoft Excel (Microsoft Corporation, Redmond, WA).

## Results

**Figure 1:**
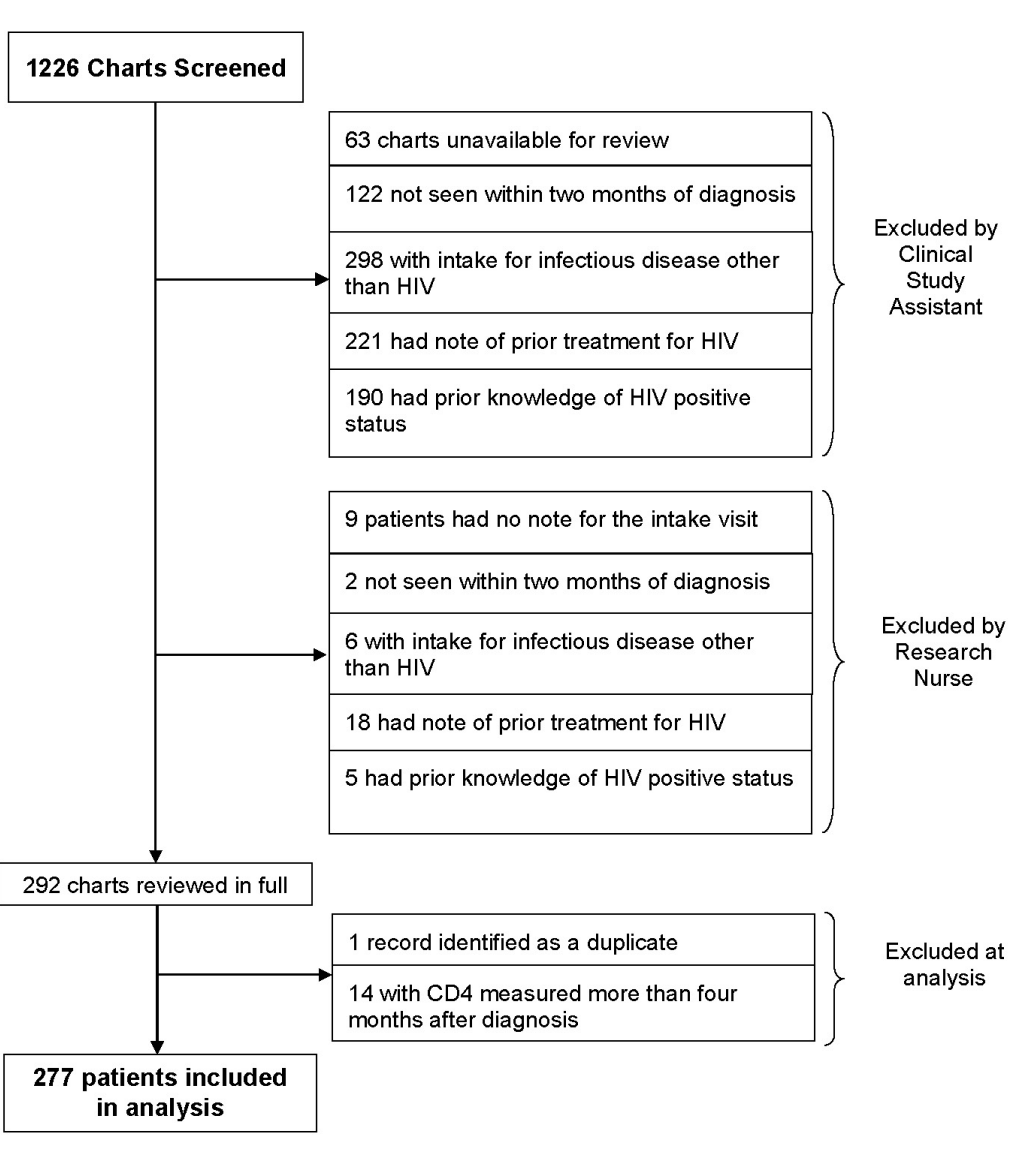
Reasons for Exclusion of Potential Study Subjects.

Of 1,226 records for patients newly attending the HIV treatment center during the study period, 277 unique patients were newly diagnosed and determined to meet study criteria. Reasons for exclusion are shown in Figure 1. Overall, included patients had a median age of 33 years (range 17 to 77 years), were 76% male, 46% African-American, and are described in Table 1. There were 143 patients identified through medical testing and 75 diagnosed by public health testing. Most medical outpatient settings referring newly diagnosed patients were either hospital affiliated primary care clinics or publically funded clinics and health centers in relatively close geographic proximity to the study site. Of those patients tested in these outpatient settings, 29 (34.5%) were privately insured, 4 (4.8%) had Medicare, 14 (16.7%) had Medicaid, 5 (6%) had some other form of public assistance, and 32 (38.1%) had no medical insurance. Other venues not classified as health care or public health testing included a health department funded HIV counseling and testing program in an emergency department [15,16] (n = 23), a publicly funded partner testing program at the region's HIV treatment center (n = 8), and universal screening by the blood bank, military, and insurance pre-screening (n = 13). Two patients were identified outside of the region and 13 were from an unknown referral source.

**Table 1:** Demographic and Risk Behavior Profile with Categorization by Testing Venue.

	Medical testing										Public health testing				Other testing											
					
	Inpatient		Outpatient		Emergency department		Unknown source		Institutional testing		Health department		Publicly funded programs		ED testing program		Identified outside the region		IDC/Partner testing		Routine screening		Unknown		Total	
	N = 37		N = 84		N = 5		N = 7		N = 10		N = 70		N = 5		N = 23		N = 2		N = 8		N = 13		N = 13		N = 277	
Age (years)	39	22–68	31.5	19–77	33	27–51	33	18–44	34	23–45	34	17–60	27	21–46	32	19–43	28.5	26–31	29	19–57	27	17–43	34	20–50	33	17–77

Black	19	51.4	49	58.3	3	60.0	1	14.3	8	80.0	21	30.0	2	40.0	12	52.2	1	50.0	1	12.5	5	38.5	5	38.5	127	45.8
White	14	37.8	30	35.7	2	40.0	6	85.7	2	20.0	41	58.6	3	60.0	11	47.8	0	0.0	7	87.5	8	61.5	8	61.5	132	47.7
Other	1	2.7	4	4.8	0	0.0	0	0.0	0	0.0	5	7.1	0	0.0	0	0.0	1	50.0	0	0.0	0	0.0	0	0.0	11	4.0
Hispanic	3	8.1	1	1.2	0	0.0	0	0.0	0	0.0	3	4.3	0	0.0	0	0.0	0	0.0	0	0.0	0	0.0	0	0.0	7	2.5

Male	30	81.1	48	57.1	4	80	5	71.4	9	90	61	87.1	3	60	22	95.7	1	50	7	87.5	12	92.3	9	69.2	211	76.2
Female	7	18.9	36	42.9	1	20	2	28.6	1	10	9	12.9	2	40	1	4.3	1	50	1	12.5	1	7.7	4	30.8	66	23.8

Injection drug use (IDU)	0	0.0	8	9.5	0	0.0	1	14.3	3	30.0	2	2.9	0	0.0	1	4.3	0	0.0	0	0.0	0	0.0	1	7.7	16	5.8
Male-male sex	13	35.1	30	35.7	3	60.0	3	42.9	4	40.0	48	68.6	2	40.0	12	52.2	0	0.0	6	75.0	7	53.8	9	69.2	137	49.5
STD diagnosis	7	18.9	22	26.2	2	40.0	0	0.0	3	30.0	24	34.3	1	20.0	7	30.4	1	50.0	1	12.5	4	30.8	1	7.7	73	26.4
Sex for drugs/money	1	2.7	2	2.4	0	0.0	0	0.0	1	10.0	0	0.0	0	0.0	1	4.3	0	0.0	0	0.0	1	7.7	1	7.7	7	2.5
Transfusion/transplant	0	0.0	2	2.4	0	0.0	1	14.3	0	0.0	1	1.4	0	0.0	2	8.7	1	50.0	0	0.0	1	7.7	0	0.0	8	2.9
Sex with an at-risk partner	3	8.1	14	16.7	0	0.0	3	42.9	0	0.0	22	31.4	1	20.0	7	30.4	0	0.0	8	100.0	1	7.7	2	15.4	61	22.0
Victim of sexual assault	1	2.7	3	3.6	0	0.0	0	0.0	0	0.0	2	2.9	0	0.0	1	4.3	2	100.0	1	12.5	0	0.0	0	0.0	10	3.6
Multiple partners	11	29.7	33	39.3	2	40.0	2	28.6	5	50.0	27	38.6	1	20.0	15	65.2	1	50.0	2	25.0	2	15.4	6	46.2	107	38.6
Heterosexual contact only	5	13.5	8	9.5	2	40.0	1	14.3	1	10.0	1	1.4	0	0.0	1	4.3	0	0.0	0	0.0	2	15.4	1	7.7	22	7.9
No risk specified	8	21.6	6	7.1	0	0.0	1	14.3	0	0.0	1	1.4	0	0.0	0	0.0	0	0.0	0	0.0	1	7.7	1	7.7	18	6.5

CD4	24	0–647	312	0–1063	293	69–731	425	12–1135	365	22–704	421	0–1299	347	0–1100	276	11–1756	352	212–492	648	12–1517	483	102–711	424	11–862	324	0–1756

Characteristics of newly diagnosed HIV patients presenting to the infectious disease center are shown with stratification by type of setting in which diagnosis occurred. The frequency and percentage of total sample is given, with the exception of age and CD4 where median value and range are provided. Patients could have multiple risk factors identified. Risk factors do not necessarily indicate the reason for testing by the setting in which the diagnosis occurred.

**Figure 2:**
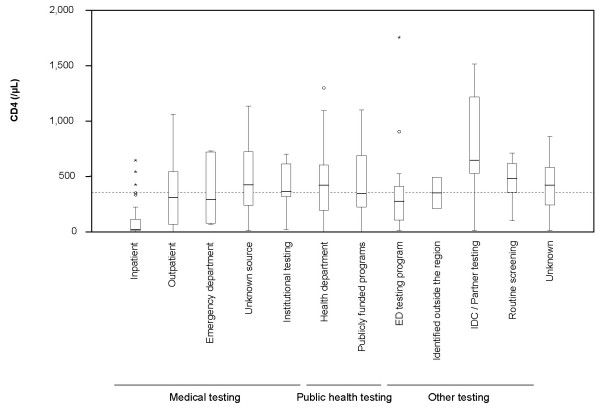
**Initial CD4 and Viral Load Measures**. Box and whisker plots of the initial CD4 count and viral load are shown with categorization by testing venue. The boxes represent the interquartile range, the bar within the boxes represents the median value. The whiskers represent the limit for outliers, shown as circles (o), defined as values falling between 1.5 and 3 box lengths from the end of the box. Cases shown as stars (*) are extreme values, defined as greater than 3 box lengths from the end of the box. The horizontal dotted line across represents a CD4 level of 350 (/μL).

Overall, there were 129 patients (46.6%) diagnosed early with a CD4 greater than 350, and 104 (37.5%) diagnosed late with a CD4 less than 200. The median values for initial CD4 count are shown in Figure 2. There were no differences in HIV viral load by site of diagnosis (data not shown). Partner testing by the IDC (median CD4 648/μL) and universal screening (median CD4 483/μL) identified patients earlier in their disease course than testing in other venues (partner testing v all others, p = 0.008 and universal screening v all others, p = 0.05). While inpatient testing contributed a large number of new diagnoses, it predominately identified patients with more advanced HIV disease (median CD4 24/μL, v all others p < 0.001).

Table 2 describes the reasons for testing for each referral source. Generally, testing because of symptoms or AIDS defining illness was more common at those venues that contributed predominately to later diagnoses. The emergency department testing program, in accordance with program design and setting, tested both for purposes of diagnosis and according to patient risk and patient self-request. Thus, amongst those with recorded reasons for testing in the emergency department; roughly half were for symptoms consistent with AIDS (7/9 had a CD 4 count < 350/μL) and half were based on risk factors or patient request (6/10 had a CD4 count > 350/μL).

**Table 2:** Reasons for HIV Testing

	Medical testing										Public health testing						Other testing						Unknown	
			
	Inpatient		Outpatient		Emergency department		Unknown source		Institutional testing		Health department		Publicly funded programs		ED testing program		Identified outside the region		IDC/Partner testing		Routine screening			
	N = 37		N = 84		N = 5		N = 7		N = 10		N = 70		N = 5		N = 23		N = 2		N = 8		N = 13		N = 13	
Not specified	11	29.7	24	28.6	2	40.0	1	14.3	4	40.0	23	32.9	4	80.0	4	18.2	0	0.0	0	0.0	1	7.7	9	69.2

Patient request	1	2.7	2	2.4	1	20.0	0	0.0	1	10.0	12	17.1	0	0.0	8	36.4	0	0.0	0	0.0	0	0.0	0	0.0
Universal screening	1	2.7	20	23.8	0	0.0	2	28.6	3	30.0	2	2.9	1	20.0	1	4.5	2	100.0	1	12.5	11	84.6	1	7.7
Reasons specified in record	24	64.9	38	45.2	2	40.0	4	57.1	2	20.0	33	47.1	0	0.0	9	40.9	0	0.0	7	87.5	1	7.7	3	23.1
*Related diagnosis*	1	4.2	4	10.5	0	0.0	0	0.0	0	0.0	1	3.0			2	22.2			0	0.0	0	0.0	0	0.0
*AIDS defining illness*	7	29.2	2	5.3	0	0.0	0	0.0	0	0.0	0	0.0			1	11.1			0	0.0	0	0.0	0	0.0
*Symptoms*	8	33.3	16	42.1	2	100.0	0	0.0	1	50.0	8	24.2			3	33.3			0	0.0	0	0.0	1	33.3
*Symptoms and related diagnosis*	0	0.0	1	2.6	0	0.0	0	0.0	0	0.0	0	0.0			0	0.0			0	0.0	0	0.0	0	0.0
*Symptoms and AIDS defining illness*	6	25.0	4	10.5	0	0.0	1	25.0	1	50.0	1	3.0			2	22.2			1	14.3	0	0.0	0	0.0
*Symptoms, related diagnosis, AIDS defining illness*	1	4.2	1	2.6	0	0.0	0	0.0	0	0.0	1	3.0			0	0.0			0	0.0	0	0.0	0	0.0
*Risk factors only*	0	0.0	6	15.8	0	0.0	2	50.0	0	0.0	18	54.5			1	11.1			5	71.4	1	100.0	2	66.7
*Risk factors with related diagnosis*	1	4.2	1	2.6	0	0.0	0	0.0	0	0.0	0	0.0			0	0.0			0	0.0	0	0.0	0	0.0
*Risk factors with symptoms*	0	0.0	2	5.3	0	0.0	1	25.0	0	0.0	3	9.1			0	0.0			1	14.3	0	0.0	0	0.0
*Risk factors, Symptoms, related diagnosis, AIDS defining illness*	0	0.0	1	2.6	0	0.0	0	0.0	0	0.0	1	3.0			0	0.0			0	0.0	0	0.0	0	0.0

The reason why the patient was tested as recorded at initial infectious disease treatment center visit are displayed and categorized by testing venue. Data are given as numbers and percentages. Reasons for testing were subcategorized as patient request, universal screening, symptoms suggestive of HIV, diagnosis potentially related to HIV, AIDS defining illness, and HIV behavioral risk factors. These categories are presented individually and in combination.

**Figure 3:**
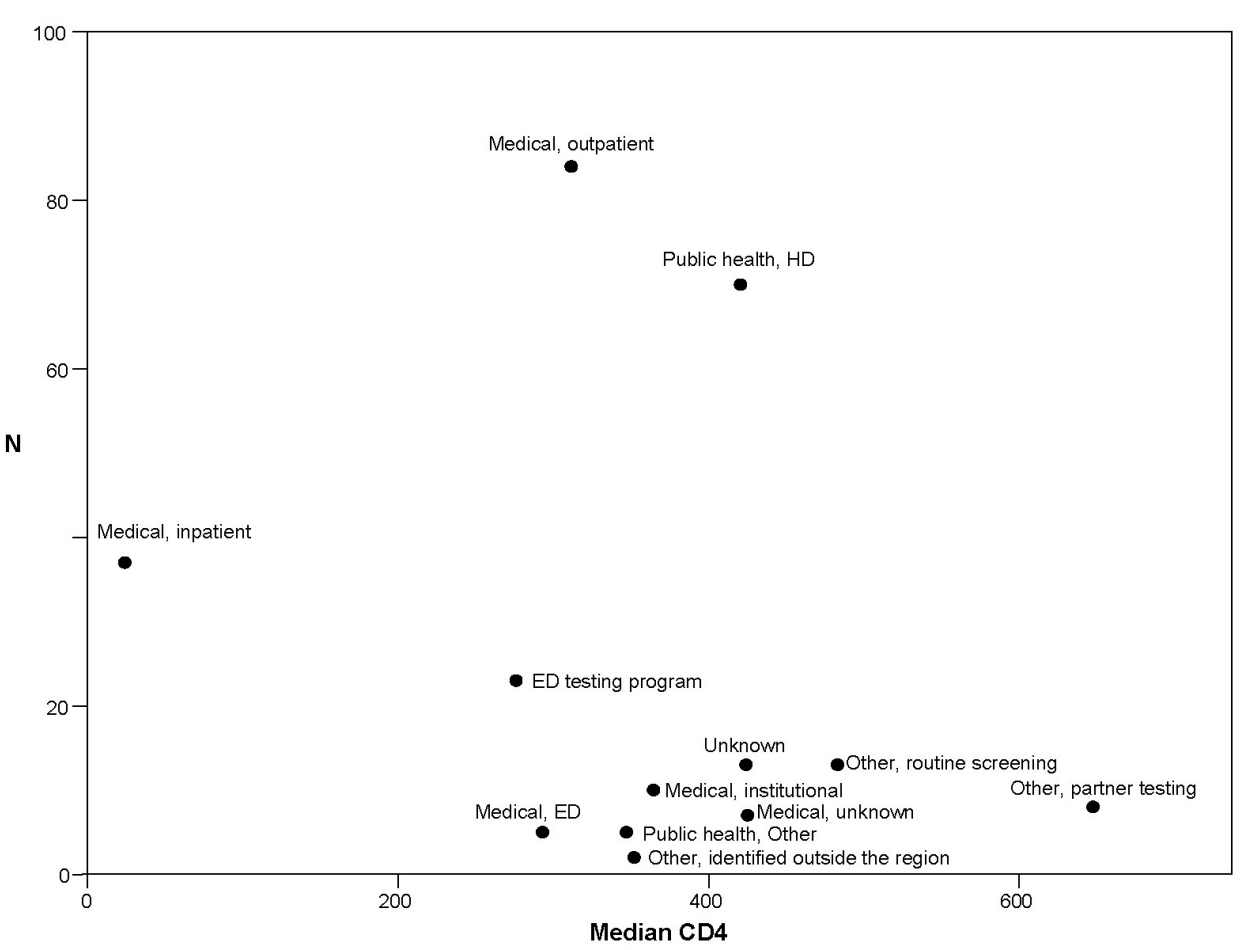
**Number of Newly Diagnosed HIV Positive Patients and Stage of Illness at Diagnosis for Regional HIV Test Venues**. The number of cases identified at each test setting is plotted against the median CD4 count at that setting. Labels are shown above or to the right of the corresponding data point. The best case scenario is a program with a high number of cases identified with a high median CD4 count (i.e. in the top right portion of the figure).

The number of new cases identified by each setting was plotted against the median CD4 count of newly diagnosed patients in Figure 3. With the exception of health department and medical outpatient testing, the results show that settings identifying patients earlier contributed fewer total cases, whereas later diagnosis by hospital testing contributed significantly to the total number of newly identified patients.

## Discussion

To our knowledge this is the first regional assessment comparing testing venues by the number of newly diagnosed patients and the stage of illness at diagnosis for patients successfully linked to subsequent care. As hypothesized, the number and timing of new HIV diagnoses are variably distributed. Our data demonstrate a mismatch between stage of diagnosis and what is optimal for public health goals; many newly identified HIV patients were diagnosed later as a result of diagnostic testing and too few were identified early enough through screening. This conceptual framework affirms the recent changes to the guidelines for testing in healthcare settings. [12] On a regional and national basis, progress towards earlier patterns of HIV diagnosis would be expected to occur if available resources were shifted for maximal effect. Screening by those venues with a track record of earlier diagnoses could be increased provided the additional resources would be effectively translated into a similar pattern of success. In settings where diagnoses are typically later, it might be possible to modify testing methods in a manner that would promote earlier diagnosis. Alternatively, resources might be shifted from screening in late diagnosing venues in favor of those providing earlier diagnoses, although diagnostic testing in such settings would presumably still be encouraged. Serial evaluations such as the one provided here or as provided by name-based HIV case surveillance could directly assess the effects of strategic planning by local, state, and national health officials. Regardless of the source of such data, monitoring new HIV diagnoses, linkage to care, and CD4 count at the time of diagnosis by testing venue should be an increasingly emphasized and utilized component of prospective surveillance efforts. With such a strategy it becomes possible to assess the extent to which patterns of resource allocation for screening and testing match patterns of early diagnosis and linkage of HIV positive patients to care without detailed investigation or reporting from every possible testing facility.

Our setting and study design was advantageous for a variety of reasons. There is a low to moderate prevalence of HIV, making results generalizable to a wide number of settings that are likely to renew and expand HIV prevention efforts after the release of the 2006 CDC guidelines for HIV testing in healthcare settings. [12] While representing an urban area of considerable size, the vast majority of HIV positive patients in the region are cared for by a single HIV treatment center. There is a highly active emergency department based HIV screening program in the area, allowing preliminary assessment of the potential importance of recently expanded CDC emphasis on emergency departments. [11,12] Our study design allowed a relatively robust sample size without the time and expense required to construct a prospective surveillance system. Also, duplication of new diagnoses by testing the same patient in different care settings was not a factor.

A primary limitation of our work is the use of records from an HIV treatment center to identify study subjects. Our analysis only indirectly equates testing venue with the testing strategies employed by those venues. Our methods did not assess the total number of tests performed or the costs of service provided for any venues. Similarly, we cannot account for diagnosed patients who never received their results or were not linked to the regional specialty care clinic. Some of these patients may have remained in, or been linked to, an alternative and appropriate patient-provider relationship. Because advanced illness would more reliably lead to contact with the HIV treatment center, our inclusion criteria would be biased in favor of those with advanced illness; the net result would be a greater representation of diagnoses arising from settings that test late in the course of illness, such as hospitals.

Another important limitation of our work is that direct assessment of screening versus diagnostic testing was not possible. Nonetheless, we have broadly grouped testing venues by the patterns of testing and patient populations that are likely to have been present during the study period. We suggest that these patterns mirror what is likely the case on a national basis. Testing by medical providers in the absence of dedicated screening efforts is likely to be primarily based on signs and symptoms of illness. This diagnostic testing has been consistently shown to lead to later diagnosis. [2,11] Not surprisingly, testing by hospitals was especially likely to identify patients with symptomatic HIV and very low initial CD4+ counts. Testing by publicly funded programs is far more likely to be a result of screening based on patient request or recognized risk factors, and accordingly leads to earlier diagnosis than diagnostic testing [2,11].

The inclusion criteria of our study may also be subject to scrutiny. We assumed all patients to be newly diagnosed if there was no direct indication to the contrary. Several patients could have tested positive months or even years prior to the positive test that ultimately led them to the infectious disease center. This too would bias our results in favor of later diagnosis. However, if there is no chart record of prior tests, patient awareness of those tests, or medical care based on those tests, then the importance of prior testing, even if it did occur, may be suspect. In this sense, a repeat positive test might still be considered a new diagnosis if it led to subsequent actions where there had been none previously.

The emergency department program was considered separately due to its unique approach. During the study period, this emergency department program was the only known healthcare setting with a large scale dedicated screening effort. Notably this program was funded by the local health department and involved health counselor screening for HIV risk. [15,16] However, the setting also provided access to patients with acute medical illness and accepted referrals from medical providers for testing based on signs and symptoms potentially suggestive of HIV. Overall, the median CD4 for the emergency department screening program was lower than anticipated, both in absolute terms and relative to other regional screening efforts. While this finding prompts many questions, it should not be interpreted as inhibitory to the increased interest in emergency department based HIV testing. Because emergency departments see both acutely ill patients and disadvantaged populations that seek emergency department care for more routine health needs, it is likely that diagnosis will result from both diagnostic testing leading to late diagnosis and screening leading to early diagnosis. Indeed, among those patients for whom a testing indication was recorded, roughly half were for symptoms consistent with AIDS and half were based on risk factors or patient request. It is also notable that, despite the support of the local health department, available resources allowed only a small proportion of the eligible emergency department population to be tested [15,16].

Patients diagnosed by the HIV treatment center had an exceptionally high median CD4 count. This testing program was targeted towards partners of HIV patients cared for at the clinic. At the time, this program was also funded by the local health department. When fully contextualized with markers of early diagnosis, the partner testing program is compelling. Our results further support the importance of partner testing programs.

There were several sources of universal screening during the study period, including insurance pre-screening, screening prior to entering military service, and blood bank surveillance. Blood bank surveillance is a unique contributor to the HIV prevention picture. The blood bank attempts to remove the majority of at-risk patients prior to donation, but then routinely tests all donations to ensure the safety of the blood supply. This is entirely different than other testing which is aimed at seeking undiagnosed HIV positive persons. It is therefore expected that the number of positive patients identified by the blood bank will be small in absolute number, but that those diagnosed will be diagnosed early and well before symptoms of illness develop, an expectation that our data supports.

Our findings should be interpreted in light of several limitations in addition to those already noted. Although we anticipated only 200 patients and actually identified 277, there were 362 of 1278 potential records that could not be located. If any missed records were randomly allocated between disease severity and referral source then it would have little effect on our primary analyses other than to reduce sample size. The low regional prevalence additionally contributes to the small sample size of some referral subgroups. Our chart review methods were rigorous, but data such as the original reason for testing or even the testing venue was frequently unavailable. There were also 14 patients excluded since they did not have a CD4+ count measured proximate to their time of diagnosis.

## Conclusion

Our data show that among patients linked to care at this referral center, many newly identified persons with HIV are diagnosed late, and the extent to which they were likely to have been diagnosed late varied by the facility in which they were diagnosed. Many of these patients were tested in healthcare settings due to a medical suspicion for HIV infection. Screening programs with demonstrated potential to identify patients with HIV early in their disease course contributed less. This is the inverse of what is desirable. This suggests that monitoring of core outcomes, including initial CD4 and linkage to care by testing venue, is a critical consideration when allocating resources. We provide a conceptual paradigm for use when resources are insufficient to maximize testing in all possible ways; settings that generally diagnose HIV positive patients late in their disease course should either modify methods to augment earlier diagnosis or be de-emphasized as a focus of coordinated screening efforts in favor of other venues. Settings that typically provide early identification should receive additional support provided that support translates to an increased absolute number of new diagnoses. Emergency department testing can contribute significantly to regional diagnosis patterns, but the extent to which that contribution is early will depend upon the strategies utilized. Markers of early diagnosis must be tracked by screening programs and policy makers to quality assure prevention efforts and coordinate prevention activities on a regional level.

## Competing interests

The authors declare that they have no competing interests.

## Authors' contributions

MSL, DAH, CJL, ATT, and CJF participated in the conception and design of the study. MSL and DAH obtained IRB approval. DAH & DLR acquired the data. Data were analyzed and interpreted by CJL, MSL, and DLR. CJL provided statistical advice and primarily analyzed the data. CJF and ATT supervised the clinical program from which the data were collected as well as providing oversight for the retrospective study. MSL drafted the manuscript and all authors contributed substantially to its revision. All authors read and approved the final manuscript. MSL takes responsibility for the paper as a whole.

## Pre-publication history

The pre-publication history for this paper can be accessed here:


